# Cochlear neural functional competence: an integrative transcriptomic module analysis of excitability, plasticity and microenvironmental support programs

**DOI:** 10.3389/fncel.2026.1776907

**Published:** 2026-02-19

**Authors:** Li Guo, Junli Wang, Ying Gao, Yuqi Feng, Baojun Wu, Xiaoyong Ren, Yang Li

**Affiliations:** Department of Otolaryngology, The Second Affiliated Hospital of Xi’an Jiaotong University, Xi’an, China

**Keywords:** cochlear implant, cytoskeletal plasticity, gene-module scoring, hearing loss, mesenchymal stromal cells, neural excitability, neurotrophin support, noise-induced hearing loss

## Abstract

**Background:**

Cochlear implants (CIs) restore hearing by directly stimulating spiral ganglion neurons (SGNs), yet auditory outcomes remain highly variable. Increasing evidence suggests that SGN survival alone incompletely predicts CI performance; instead, transcriptional programmes governing neuronal excitability/synaptic transmission, structural plasticity, trophic–metabolic support and injury/inflammation may better reflect neural functional competence.

**Methods:**

We re-analysed publicly available cochlear transcriptomic datasets spanning development, adulthood and injury/degeneration. Primary resources included developmental FACS RNA-seq of hair cells and surrounding tissue, adult inner- and outer-hair-cell microarrays, and a noise-induced hearing loss (NIHL) RNA-seq cohort following mesenchymal stromal cell (MSC) therapy. We additionally analysed independent injury/degeneration datasets, including spatial transcriptomics of spiral ganglion regions after noise exposure and spiral ganglion RNA-seq after aminoglycoside-induced deafening. Guided by cochlear neuroscience literature and functional enrichment, we assembled gene modules for excitability, plasticity, trophic/metabolic support and injury/inflammation. Module activity was quantified using within-dataset standardized scores from normalized expression, avoiding cross-platform merging. We derived a Cochlear Neural Functional Competence (CNFC) score (Excitability + Trophic − Injury) and assessed robustness using a minimal 24-gene panel. External validation was performed in an independent purified SGN dataset, and CNFC was benchmarked against transcriptome-wide principal components.

**Results:**

Developmental maturation was characterized by increasing excitability-associated transcripts alongside down-regulation of actin/cytoskeletal remodelling components. Adult hair cells displayed distinct trophic signatures. In the NIHL model, MSC therapy was associated with transcriptional suppression of excitatory receptor and channel genes, consistent with a shift in the injury/inflammation–excitability balance, although functional consequences remain to be established. Importantly, in independent injury/degeneration datasets, CNFC decreased in spiral ganglion neuronal regions after noise exposure and in deafened spiral ganglion. Across datasets, CNFC captured coherent trends and remained highly correlated with full-module scoring when reduced to the 24-gene panel.

**Conclusion:**

CNFC is a transparent, hypothesis-generating framework for summarizing cochlear neuronal functional state from transcriptomic data, complementing traditional survival metrics. By prioritizing interpretable modules and standardized within-dataset scoring, CNFC supports cross-study integration and highlights candidate programmes for mechanistic testing.

## 1 Introduction

Sensorineural hearing loss is a leading cause of disability worldwide (World Health Organization, 2025), and cochlear implantation is the most effective intervention for severe-to-profound cases. By bypassing damaged hair cells, cochlear implants (CIs) deliver patterned electrical stimulation to spiral ganglion neurons (SGNs) and downstream auditory circuits. Despite continuous advances in electrode design, sound-processing strategies and surgical techniques, speech perception outcomes vary widely across CI recipients, and this variability is only partially explained by clinical covariates such as age, duration of deafness or residual hearing (Bartholomew et al., 2024; Finley et al., 2008; Holden et al., 2016).

A common assumption is that CI benefit is primarily determined by the number of surviving SGNs. However, the correlation between spiral ganglion cell counts and speech perception is variable and overall modest, implying that anatomical survival alone does not capture the neural capacity to encode and transmit temporally precise information (Cheng and Svirsky, 2021). Neural response properties such as refractoriness, accommodation and spike-rate adaptation further shape temporal coding under high-rate CI stimulation (Boulet et al., 2016). We therefore distinguish between (i) structural survival (persistence of neuronal cell bodies) and (ii) functional competence, defined as the molecular and cellular state enabling fast firing, reliable synaptic transmission and the ability to adapt to chronic electrical stimulation. This framing is consistent with growing recognition that synaptic disconnection and “hidden” neural injury can occur even when sensory cells survive, and may degrade auditory coding without proportionate changes in audiometric thresholds (Kujawa and Liberman, 2009; Lin et al., 2011; Liberman and Kujawa, 2017).

During development, auditory neurons acquire high-frequency firing by up-regulating fast voltage-gated channels and refining synaptic machinery, while simultaneously shutting down growth-associated cytoskeletal programmes (Wang et al., 1998; Adamson et al., 2002; Hossain et al., 2005; Browne et al., 2017). Transcriptomic studies also highlight marked sensory-neuron diversity in the auditory periphery and activity-dependent shaping of afferent identities (Shrestha et al., 2018; Petitpré et al., 2018). Spatial differences in intrinsic firing and channel composition also align with the tonotopic organization of the spiral ganglion (Adamson et al., 2002; Nayagam et al., 2011; Reijntjes and Pyott, 2016). After hair-cell loss, deafferentation, or noise trauma, neurons may persist structurally yet regress functionally through transcriptional changes, synaptic loss and microenvironmental remodeling (Kujawa and Liberman, 2009; Lin et al., 2011; Liberman and Kujawa, 2017). Cell-type-resolved transcriptomic atlases of acoustic trauma further emphasize coordinated glial and immune responses in the injured cochlea (Milon et al., 2021). Importantly, the cochlear microenvironment—including hair cells, supporting cells, glia and immune components—supplies trophic factors, extracellular matrix cues and metabolic buffering that can either sustain or erode neural competence (Ramekers et al., 2012; Wan et al., 2014; Suzuki et al., 2016; Wang and Green, 2011).

Here, we performed an integrative reanalysis of three primary publicly available mouse cochlear transcriptomic resources spanning developmental maturation, adult hair-cell programmes and post-trauma intervention, and complemented these with an independent purified spiral ganglion neuron dataset for external validation. Rather than emphasising single-gene findings, we organised results into programme-level modules—excitability, plasticity, trophic/metabolic support and injury modulation—that can be linked to clinically actionable neuroprosthetic hearing phenotypes.

## 2 Materials and methods

### 2.1 Overall study design and dataset selection

**Figure 1:**
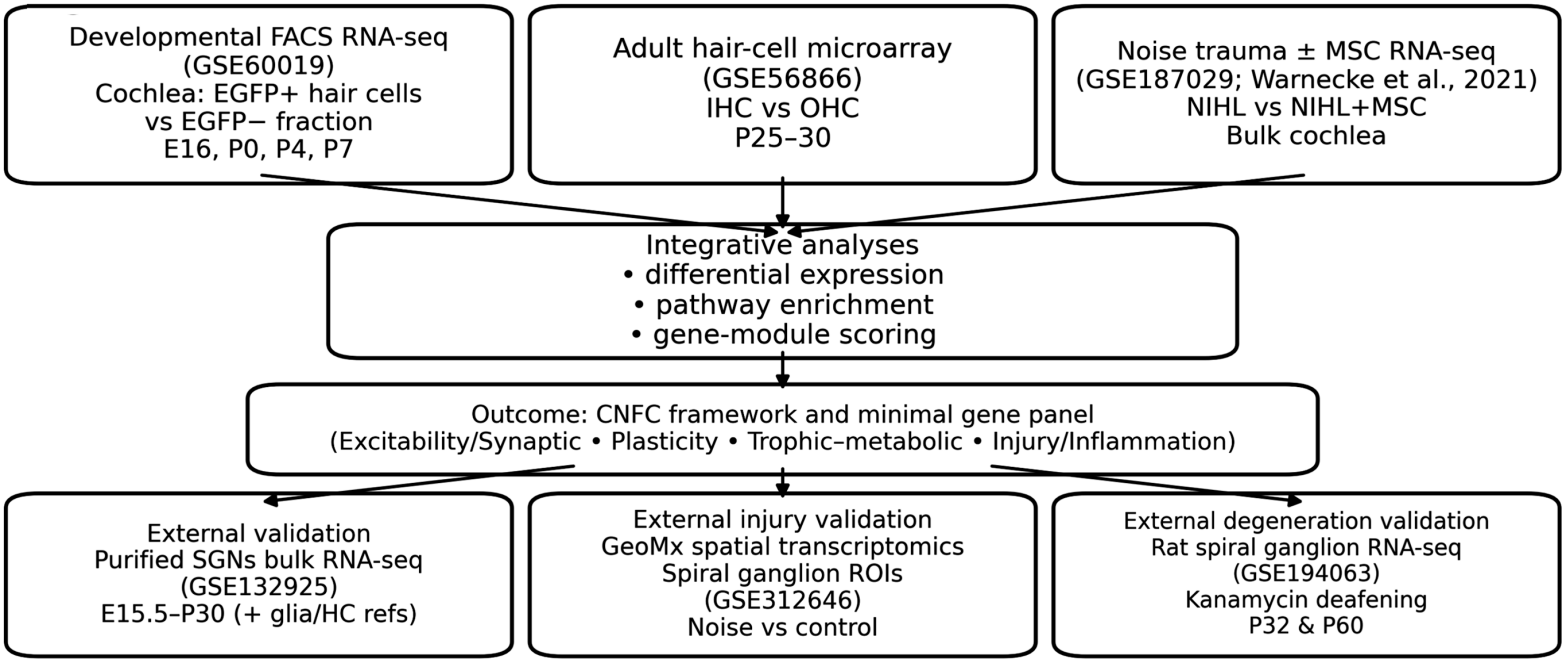
Study overview and datasets. Schematic of the integrative transcriptomic strategy and data resources. Public datasets included: (i) Developmental cochlear FACS RNA-seq (GSE60019; EGFP+ hair cells vs. EGFP− non-hair cells across E16, P0, P4, and P7), (ii) adult hair-cell microarray (GSE56866; inner hair cells vs. outer hair cells at P25–30), (iii) noise trauma ± mesenchymal stromal cell (MSC) therapy RNA-seq study (Warnecke et al., 2021; bulk cochlea; sound trauma vs. sound+MSC; re-analysed from the deposited TMM-normalized count matrix, GSE187029), (iv) external validation bulk RNA-seq of purified spiral ganglion neurons across five ages with glial and hair-cell reference samples (GSE132925) (Li et al., 2020), and additional independent injury/degeneration validation datasets: (v) GeoMx spatial transcriptomics of spiral ganglion regions after noise exposure (GSE312646) and (vi) rat spiral ganglion RNA-seq after kanamycin-induced deafening (GSE194063). Analyses comprised differential expression, pathway enrichment and gene-module scoring to derive an integrated cochlear neural functional competence (CNFC) framework and a minimal gene signature for reuse.

We selected three primary publicly available mouse cochlear transcriptomic resources spanning development and injury/repair: (i) a developmental FACS RNA-seq dataset (GSE60019) profiling Atoh1-EGFP+ hair cells and the surrounding EGFP− fraction at E16, P0, P4 and P7 (Scheffer et al., 2015); (ii) adult inner- and outer-hair-cell microarrays (GSE56866) capturing sensory-epithelial trophic signalling programmes (Liu et al., 2014); and (iii) an NIHL RNA-seq dataset following mesenchymal stromal cell (MSC) therapy (GSE187029) (Warnecke et al., 2021). To test generalisability beyond the datasets used for module construction, we additionally analysed: (iv) an independent purified spiral ganglion neuron (SGN) bulk RNA-seq dataset spanning development (GSE132925), and (v) two independent injury/degeneration resources—GeoMx spatial transcriptomics of spiral ganglion regions after noise exposure (GSE312646) and rat spiral ganglion RNA-seq after kanamycin-induced deafening (GSE194063). Because these datasets were generated on heterogeneous platforms (bulk RNA-seq, FACS RNA-seq, microarrays and GeoMx) and across species, we did not merge expression matrices across studies; instead, all analyses were performed within each dataset (and within GeoMx segments), and cross-study comparisons are presented as standardized scores and effect sizes rather than absolute quantitative equivalence. Figure 1 summarizes the study design.

### 2.2 Developmental FACS RNA-seq processing (GSE60019)

Processed expression tables were downloaded from GEO and analysed separately for the EGFP+ (hair-cell) and EGFP− (non-hair-cell) fractions at E16, P0, P4 and P7. For descriptive gene-level plots and fold-change summaries, we used library-size-normalized expression (counts-per-million, CPM) and log2-transformed values. For module scoring, expression values were standardized within the developmental dataset (*z*-scored across stages within each fraction) to emphasize temporal trends independent of platform-specific scaling.

### 2.3 Adult inner/outer hair-cell microarray processing (GSE56866)

Normalized microarray expression values were downloaded from GEO. Probe sets were mapped to gene symbols using the annotation provided with the dataset; when multiple probe sets mapped to the same gene, values were collapsed by the median. Differential expression between inner hair cells (IHCs) and outer hair cells (OHCs) was assessed on log2 expression values. For module scoring, gene expression was standardized within the microarray dataset (*z*-scored across the seven samples).

### 2.4 NIHL + MSC dataset processing (GSE187029)

**Figure 2:**
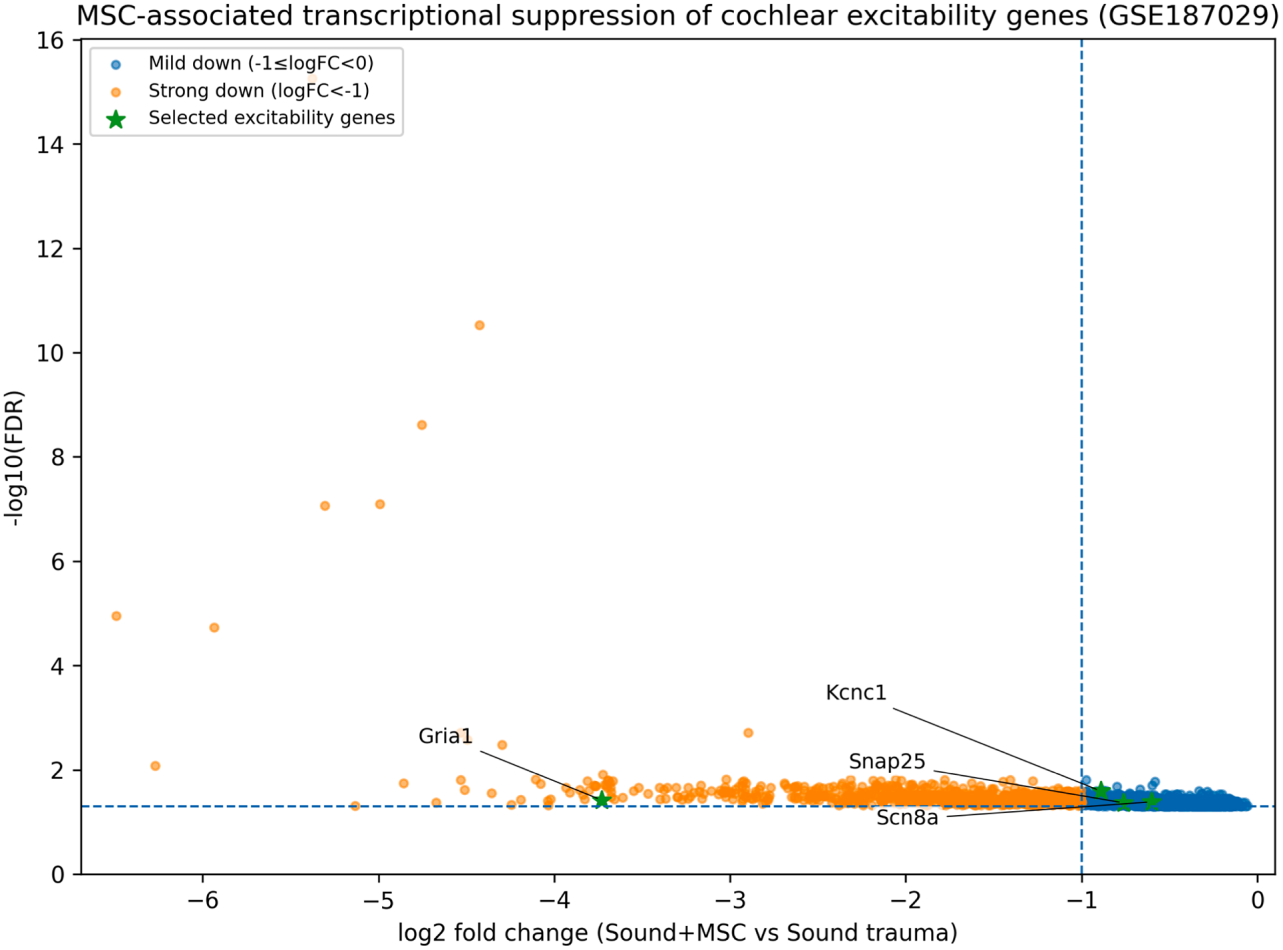
NB-GLM re-analysis of the NIHL + MSC dataset reveals coordinated suppression of excitability-associated programs. Volcano plot showing gene-level differential expression (negative binomial GLM; log2FC for MSC vs. untreated NIHL; Benjamini–Hochberg FDR). Dashed lines indicate |log2FC| = 1 and FDR = 0.05. Genes belonging to the excitability, plasticity, and trophic/metabolic modules are highlighted (symbols; see legend), and representative excitability genes are labeled. Module-level effect sizes and gene-set test statistics are provided in Supplementary Table S1.

For the NIHL RNA-seq study (Warnecke et al., 2021), we re-analysed the TMM-normalized gene count matrix provided as a GEO (Supplementary File) (GSE187029_msc_experiment_tmm_raw_counts.txt.gz), comprising whole-cochlea RNA from noise-exposed mice with or without MSC therapy (*n* = 3 per group). Genes with low expression were filtered (CPM > 1 in at least two samples). Differential expression (MSC vs. untreated NIHL) was re-computed using a negative binomial generalized linear model (log link) with a group indicator and a library-size offset (log total counts per sample). A common dispersion parameter (*α*) was estimated across expressed genes using a method-of-moments approach (median *α* = 0.067). Two-sided Wald *p*-values were adjusted using Benjamini–Hochberg FDR. These gene-level statistics were used for downstream module summaries and visualization (Figure 2).

#### 2.4.1 GeoMx spatial transcriptomics after noise exposure (GSE312646)

We analysed the GeoMx DSP expression table from a study profiling spiral ganglion stress responses after noise exposure (Yu et al., 2025). The table contains segmented regions of interest (ROIs) for spiral ganglion neurons (‘Neu’) and supporting cells (‘Supp’) from control and noise-exposed cochleae. Because this platform assays a targeted gene panel, module scores were computed using the intersection between the assayed genes and our full CNFC gene sets (Excitability *n* = 8, Plasticity *n* = 8, Trophic/Metabolic *n* = 7, Injury/Inflammation *n* = 4; Supplementary Table S1). Within each segment type, expression values were log2(x + 1) transformed and standardized per gene (*z*-score). Module scores were defined as the mean *z*-score across module genes, and CNFC was computed as Excitability + Trophic/Metabolic − Injury/Inflammation. Noise vs. control differences were assessed using Welch’s *t*-tests.

#### 2.4.2 Rat spiral ganglion RNA-seq after kanamycin deafening (GSE194063)

We used the normalized read-count matrix deposited with a rat spiral ganglion degeneration study (Gansemer et al., 2024), including hearing and kanamycin-deafened animals sampled at P32 and P60. One P32 hearing replicate is not present in the deposited normalized matrix (excluded as a transcriptomic outlier in the original study); analyses were performed on the remaining samples. Normalized expression values were log2(x + 1) transformed and standardized per gene. Module and CNFC scores were calculated as above (using full gene sets). Effects of deafening on CNFC were tested with a linear model including age as a covariate (CNFC ~ group + age) and with within-age comparisons.

### 2.5 Module construction, scoring and cross-study comparability

Four coherent transcriptional modules were defined: (i) excitability/synaptic transmission, (ii) plasticity/neurite growth, (iii) trophic–metabolic support and (iv) injury/inflammation. Seed genes were nominated from cochlear and SGN literature and then expanded/verified using functional enrichment results (Section 2.6). To quantify module activity within each dataset, we used a simple per-sample gene-set scoring approach conceptually related to GSVA and GSEA (Hänzelmann et al., 2013; Subramanian et al., 2005): gene expression was normalized and log2-transformed as appropriate for each platform, standardized per gene within the dataset (*z*-score across samples), and module scores were computed as the mean *z*-score across genes in the module. Because datasets were generated on heterogeneous platforms and in different contexts, we do not merge expression matrices across studies; cross-study comparisons are therefore presented as within-dataset standardized module scores and standardized effect sizes (e.g., Cohen’s d) rather than absolute quantitative equivalence. For external validation plots in purified SGNs (GSE132925), we additionally report raw module expression as the median log2(CPM + 1) across module genes; median- versus mean-based aggregation yielded highly correlated CNFC trajectories (Supplementary Table S1). The composite CNFC score was defined as Excitability + Trophic/Metabolic − Injury/Inflammation; plasticity was tracked as an independent axis because it can reflect both developmental consolidation and injury-driven regrowth programmes (see Tables 1, 2).

**Table 1:** Representative developmental switches in cochlear hair-cell (EGFP+) and non-hair-cell (EGFP−) fractions (GSE60019).

Gene	Module	E16 EGFP+	P7 EGFP+	P7/E16 EGFP+	E16 EGFP−	P7 EGFP−	P7/E16 EGFP−
Cfl1	Plasticity (actin remodelling)	64,010	13,857	0.22	53,858	43,498	0.81
Cfl2	Plasticity (actin remodelling)	8,997	1,508	0.17	5,094	7,140	1.40
Syt4	Developmental/synaptic window	1,206	65	0.05	952	174	0.18
Hmga2	Developmental regulator	8,248	373	0.05	28,748	279	<0.01
Cdk5	Synaptic maintenance	46	111	2.41	32	75	2.34
Cdk5r1	Synaptic maintenance (activator)	12,111	609	0.05	838	671	0.80
Kcnc1	Excitability (fast K + channel)	38	6	0.16	3	7	2.64
Gria1	Excitability (AMPA receptor)	14	0	0.00	74	635	8.56
Mcl1	Survival/stress buffering	4,184	1,406	0.34	3,798	5,490	1.45
Sox2	Supporting-cell programme	2,313	195	0.08	787	1,149	1.46

Expression values in this table are taken from processed, library-size-normalized tables and are shown for descriptive purposes. All comparisons across conditions/stages were performed on normalized log2 expression values (CPM-based for RNA-seq; log2-normalized microarray expression), as described in Methods.

**Table 2:** Candidate gene programmes contributing to cochlear neural functional competence and potential translational relevance.

Module	Representative genes	Evidence across datasets	Potential CI relevance
Excitability & synaptic transmission	Gria1, Grin2a/Grin2c, Kcnc1, Scn8a, Gabra6	Developmental increase of Gria1 and Kcnc1 in EGFP- fraction (GSE60019); strong down-regulation of Grin2a/Grin2c, Kcnc1, Scn8a and Gabra6 with MSC therapy after NIHL	May influence temporal coding, stimulation thresholds and susceptibility to excitotoxic stress during chronic electrical stimulation
Cytoskeletal plasticity & neurite growth	Cfl1, Cfl2, Hmga2 (and related actin regulators)	Marked postnatal decline of Cfl1/Cfl2, Hmga2 and Syt4 in hair-cell fraction; modest decline and reshaping of plasticity signatures in EGFP- fraction	Reduced intrinsic growth competence may limit neurite extension and electrode-neuron coupling; targets for regeneration-oriented interventions
Sensory-epithelial trophic support	Ntf3, Gdnf, Bdnf, Spp1	IHC-biased expression of Ntf3 and Gdnf in adult hair-cell microarray (GSE56866); Bdnf and Spp1 detected in both hair-cell types	Microenvironmental trophic status may protect SGNs and synapses, supporting functional competence even after hair-cell loss
Metabolic resilience / oxidative stress buffering	Mitochondrial and proton-pump genes (e.g., Sdhd, Atp6v1h); anti-apoptotic genes (e.g., Mcl1)	High metabolic gene expression in hair cells; Mcl1 maintained or increased in EGFP- fraction during development	Energy-demanding auditory signalling may render the cochlea vulnerable to oxidative injury; metabolic resilience may affect long-term CI benefit
Injury-response modulation	Stress/inflammation and extracellular matrix genes (dataset-dependent)	MSC therapy after NIHL is associated with broad transcriptional suppression of neural signalling genes; original publication reports additional repair-associated pathways	Interventions that rebalance inflammation, matrix remodelling and excitability may protect neural competence and improve outcomes
CNFC scoring / minimal panel	24-gene CNFC panel (Table 3)	Minimal-panel composite scores correlate with full-module scores across developmental and adult datasets (Figure 3B; Supplementary Table S1).	Provides a reusable, compact starting point for targeted molecular assays (e.g., qPCR/ddPCR or targeted RNA panels) to profile functional competence in future cohorts.

### 2.6 Functional enrichment analysis

Gene Ontology (GO) and KEGG pathway enrichment analyses were performed using g:Profiler with g:SCS multiple-testing correction (Raudvere et al., 2019). Enrichment queries used the *Mus musculus* gene universe and were applied to differentially expressed genes in each dataset to provide an independent functional check on the curated module definitions and to transparently document pathway support for module membership. GO and KEGG annotations were referenced from the Gene Ontology resource and KEGG pathway databases, respectively (The Gene Ontology Consortium, 2021; Kanehisa and Goto, 2000).

### 2.7 Cell-type signature scoring in the EGFP− fraction

To estimate the changing cellular composition of the EGFP− fraction across development, we computed three cell-type signature scores (neuronal, supporting-cell and immune) as the mean *z*-scored expression of marker genes curated from published cochlear atlases. Signature scores were compared across E16, P0, P4 and P7 and related to the excitability module to test whether excitability trends could be explained by changing neuronal enrichment.

### 2.8 Minimal CNFC gene panel

**Figure 3:**
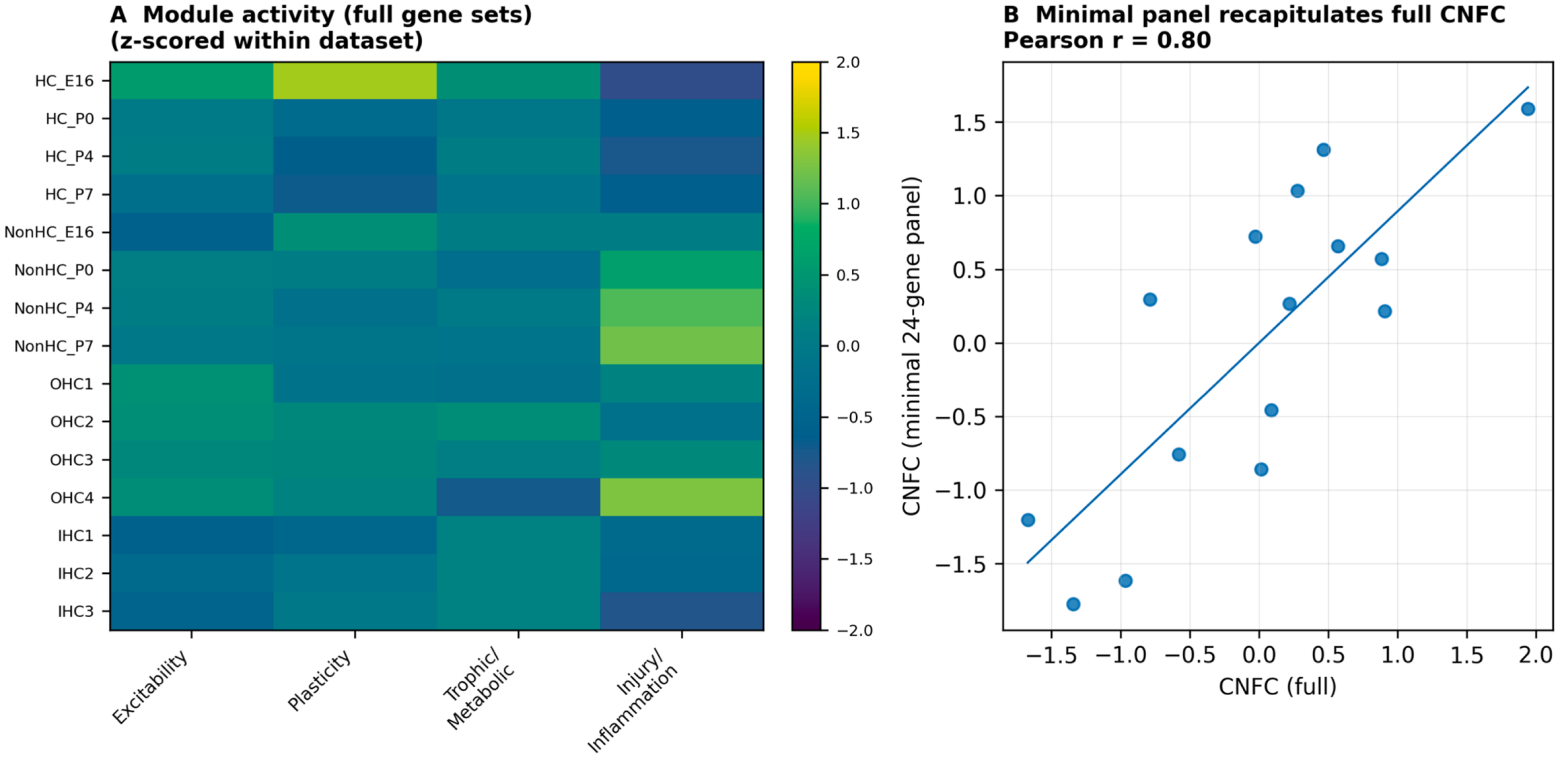
Cross-dataset module scoring and a minimal CNFC gene signature. **(A)** Heatmap of standardized module scores (full gene sets) across the developmental FACS dataset and adult hair-cell microarrays. **(B)** Correlation between CNFC computed using the full gene sets versus the minimal 24-gene panel across all samples (Pearson *r* shown).

To facilitate reuse across platforms and studies, we defined a minimal 24-gene panel by selecting highly referenced, functionally representative genes with consistent detection across datasets (6 excitability, 5 plasticity, 7 trophic/metabolic and 6 injury/inflammation genes; Table 3). Minimal-panel module scores and CNFC were computed using the same procedures as the full modules. Agreement between full and minimal CNFC scores was assessed by correlation across all samples (Figure 3B).

**Table 3:** Minimal 24-gene CNFC panel for reusable module scoring.

Module	Gene	Rationale (example function)
Excitability	Kcnc1	Kv3.1; supports high-frequency firing
Excitability	Scn8a	Nav1.6; action potential initiation/propagation
Excitability	Gria1	AMPA receptor; fast excitatory transmission
Excitability	Grin2a	NMDA receptor subunit; synaptic signalling/plasticity
Excitability	Grin2c	NMDA receptor subunit; excitatory synaptic signalling
Excitability	Snap25	SNARE protein; synaptic vesicle exocytosis
Plasticity	Cfl1	Cofilin-1; actin remodelling
Plasticity	Cfl2	Cofilin-2; actin remodelling
Plasticity	Hmga2	Growth-associated chromatin factor; developmental plasticity
Plasticity	Syt4	Synaptotagmin-4; activity-linked synaptic function
Plasticity	Cdk5	Cyclin-dependent kinase 5; neurite/synapse maintenance
Trophic/Metabolic	Ntf3	Neurotrophin-3; SGN survival/synaptic maintenance
Trophic/Metabolic	Gdnf	Neurotrophic factor; neuronal support
Trophic/Metabolic	Bdnf	Neurotrophic factor; neuronal support/plasticity
Trophic/Metabolic	Spp1	Osteopontin; extracellular support signalling
Trophic/Metabolic	Ntrk2	TrkB receptor; neurotrophin signalling
Trophic/Metabolic	Sdhd	Mitochondrial complex II; metabolic resilience
Trophic/Metabolic	Atp6v1h	V-ATPase subunit; energy/acidification homeostasis
Injury/Inflammation	S100a8	Inflammation marker; innate immune activation
Injury/Inflammation	S100a9	Inflammation marker; innate immune activation
Injury/Inflammation	Ccl3	Chemokine; immune recruitment
Injury/Inflammation	Ccl4	Chemokine; immune recruitment
Injury/Inflammation	Lcn2	Injury/inflammation marker; stress response
Injury/Inflammation	Mmp9	Matrix metalloproteinase; ECM remodelling/inflammation

Full module gene lists and per-sample module scores are provided in Supplementary Table S1.

### 2.9 Unbiased transcriptome-level validation and weight sensitivity

**Figure 4:**
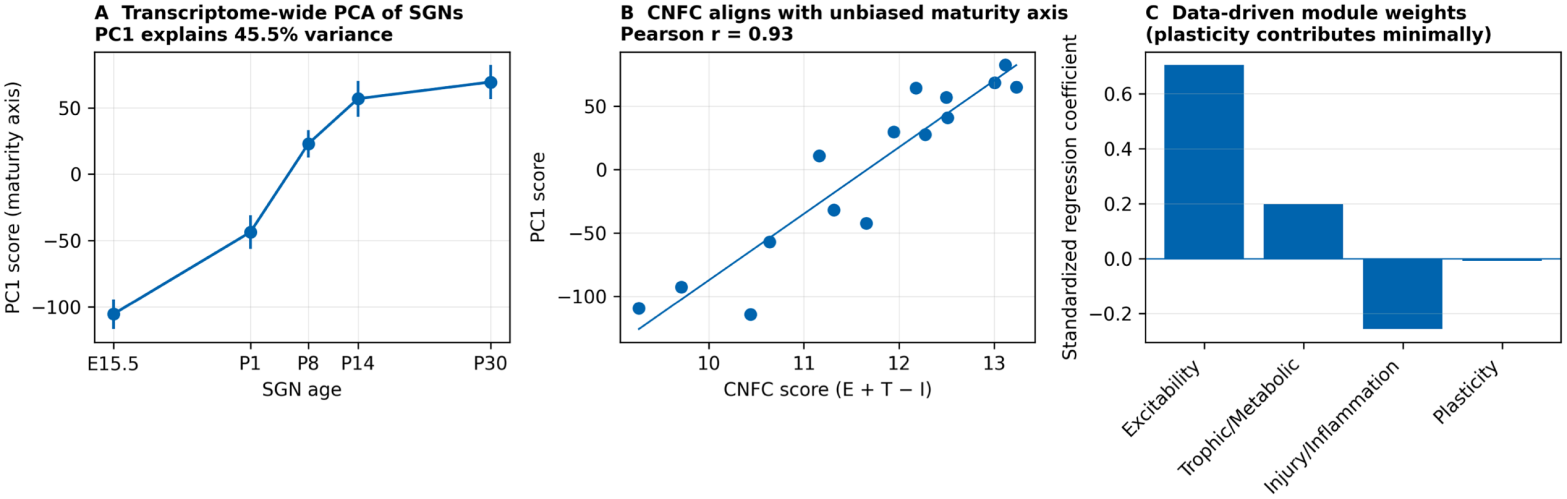
Unbiased transcriptome-wide validation of CNFC in purified SGNs (GSE132925). **(A)** Principal-component analysis (PCA) of transcriptome-wide SGN expression identifies a dominant maturity axis (PC1) across ages. **(B)** CNFC correlates strongly with PC1 (Pearson *r* shown), indicating that the curated CNFC composite aligns with an unbiased transcriptional trajectory. **(C)** Data-driven regression coefficients estimating module weights for predicting PC1 highlight a dominant contribution of excitability and trophic/metabolic support and a minimal contribution of plasticity.

To benchmark CNFC against an unbiased transcriptional summary, we performed principal-component analysis (PCA) on log2(CPM + 1) expression for purified SGNs (GSE132925) after filtering low-variance genes. We tested whether CNFC and individual module scores aligned with the dominant transcriptomic maturity axis (PC1) by correlation analysis (Figure 4). To address concerns about equal module weighting, we also estimated data-driven weights by multivariate regression predicting PC1 from standardized module scores and compared weighted versus unweighted CNFC (Supplementary Table S1).

## 3 Results

### 3.1 Developmental up-regulation of excitability/synaptic programmes in the non-hair-cell fraction

**Figure 5:**
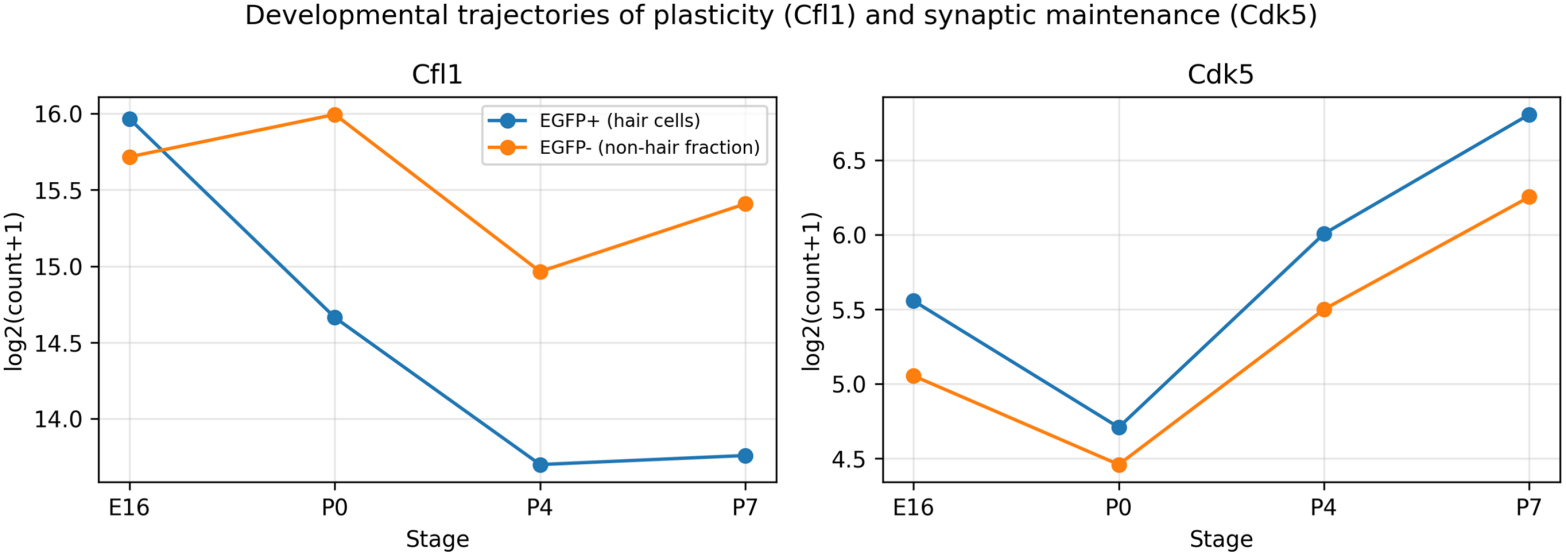
Developmental trajectories of representative plasticity and synaptic maintenance genes. Time-course expression (log2 transformed counts) of *Cfl1* and *Cdk5* in EGFP+ and EGFP- cochlear fractions from E16 to P7. The decline of *Cfl1* in EGFP+ hair cells contrasts with increasing synaptic maintenance demands reflected by *Cdk5* up-regulation.

In the EGFP− fraction of the developing cochlea (GSE60019), excitability and synaptic transmission genes increased from embryonic to early postnatal stages, consistent with maturation of auditory neuronal properties. Representative examples included ion channels (e.g., Kcnc1, Scn8a), glutamate receptor components (e.g., Gria1) and synaptic machinery (e.g., Snap25; Figure 5). Importantly, all developmental comparisons were performed on normalized expression (CPM, log2-transformed) rather than raw counts to account for sequencing depth.

**Figure 6:**
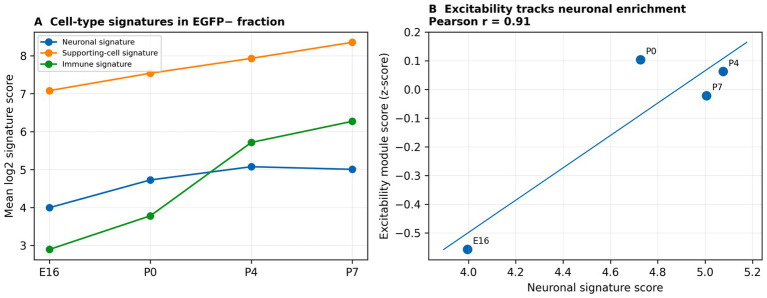
Cell-type signature scoring supports a neural component of the EGFP− excitability programme. **(A)** Developmental changes in neuronal, supporting-cell and immune signature scores in the EGFP− fraction. **(B)** Excitability module score tracks the neuronal signature score across stages (Pearson *r* shown), supporting partial contribution of changing neuronal enrichment to excitability trajectories.

To test whether these excitability trends could reflect changing cell composition within the EGFP− fraction, we scored neuronal, supporting-cell and immune marker signatures. Neuronal signature scores increased in parallel with the excitability module (Figures 6A,B), suggesting that both transcriptional maturation and increasing neuronal enrichment contribute to the observed excitability trajectory.

### 3.2 Developmental consolidation of cytoskeletal/plasticity programmes in the hair-cell fraction

**Figure 7:**
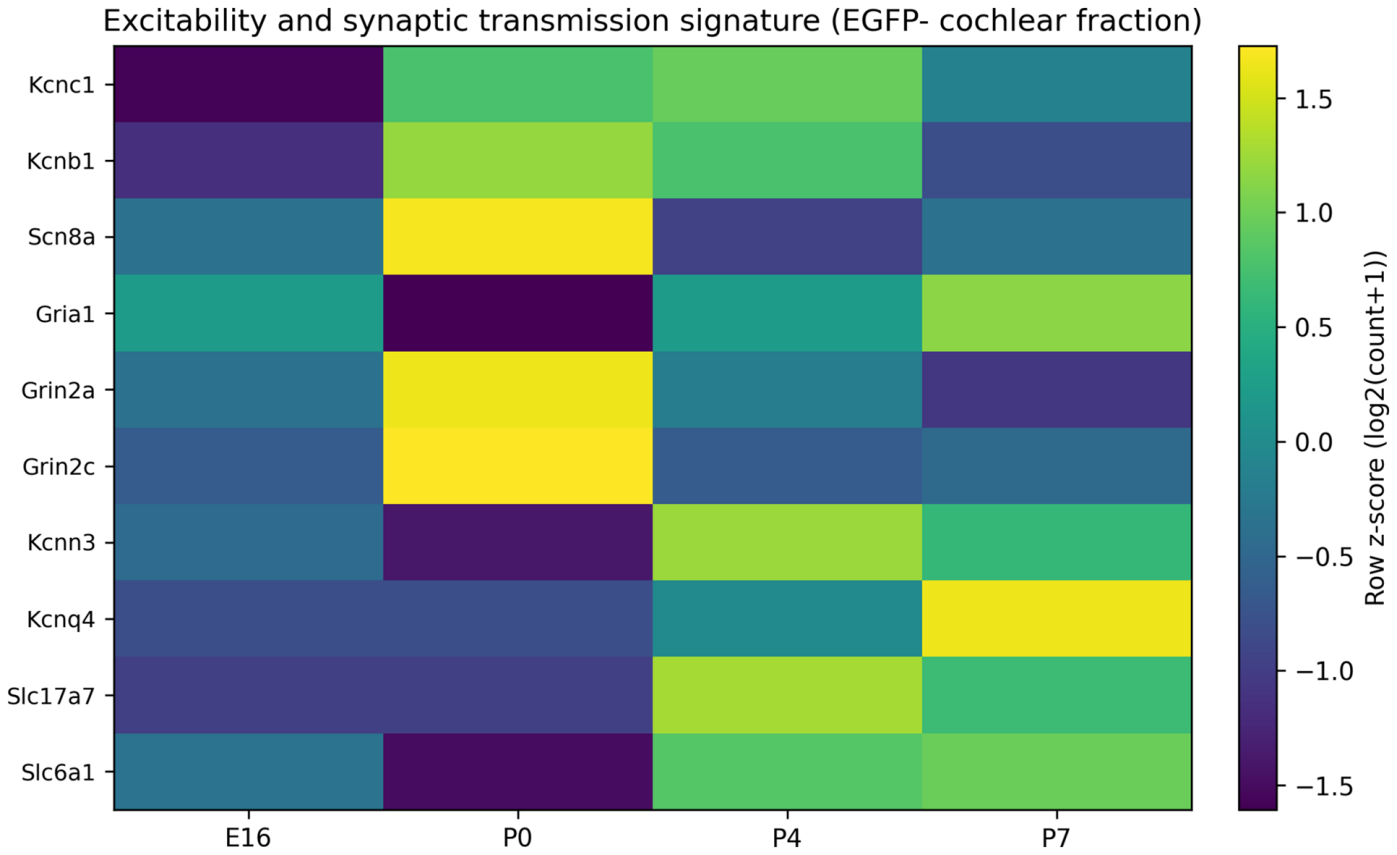
Maturation of a neural excitability signature in the EGFP-fraction. Heatmap of representative excitability and synaptic-transmission genes (e.g., *Gria1*, *Kcnc1*, selected voltage-gated channels, and receptor genes) across E16, P0, P4, and P7 within the EGFP- fraction. Genes are scaled by row (*z*-score) to highlight relative developmental changes.

In the EGFP+ hair-cell fraction, genes associated with actin remodelling and structural dynamics (e.g., Cfl1/Cfl2) declined across early postnatal development (Figure 7). While Cfl1/Cfl2 are representative actin regulators, we interpret this pattern conservatively as consolidation of developmental growth/remodelling programmes rather than a direct proxy for regenerative potential, which can involve distinct injury-induced gene programmes.

### 3.3 Adult hair-cell trophic specialisation highlights sensory-epithelial support pathways

**Figure 8:**
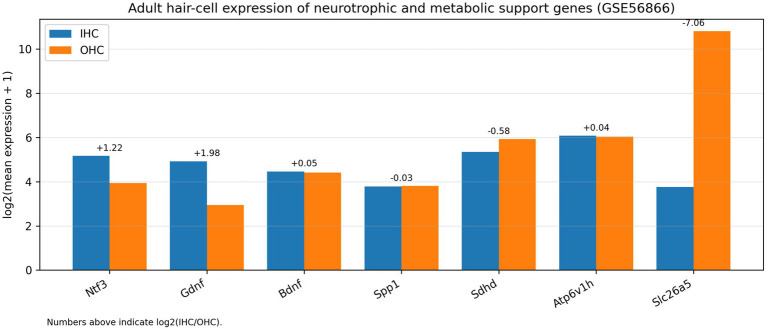
Adult hair-cell trophic programs supporting cochlear neurons. Representative trophic and metabolic genes expressed in adult inner hair cells (IHCs) versus outer hair cells (OHCs). Expression is shown as log2(mean normalized microarray expression + 1) across biological replicates (*n* = 3 IHC, *n* = 4 OHC).

In adult hair-cell microarrays (GSE56866), inner and outer hair cells displayed distinct expression of trophic ligands, extracellular matrix regulators and metabolic mediators (Figure 8), supporting the idea that the sensory epithelium provides an active microenvironment shaping neural competence.

### 3.4 MSC therapy is associated with concerted suppression of excitability-associated transcripts in NIHL

We re-analysed the NIHL + MSC cohort (GSE187029) from the TMM-normalized count matrix using a negative binomial GLM (Methods 2.4). Relative to untreated noise-exposed cochleae, MSC-treated cochleae showed strong down-regulation of the excitability module (mean log2FC = −2.59 across 12 mapped genes; directional Stouffer gene-set *p* = 6.8 × 10–128; Supplementary Table S1). This included large shifts in receptor and synaptic genes such as Gabra6 (log2FC = −7.07, FDR = 1.7 × 10–39), Grin2a (log2FC = −5.13, FDR = 1.8 × 10–19), Grin2c (log2FC = −3.76, FDR = 2.4 × 10–5), Gria1 (log2FC = −1.75, FDR = 6.3 × 10–20), Slc17a7 (log2FC = −2.08, FDR = 2.6 × 10–3), and synaptic release machinery (e.g., Syt1 log2FC = −3.38, FDR = 8.4 × 10–37; Snap25 log2FC = −2.69, FDR = 1.2 × 10–17) (Figure 2). Plasticity and trophic/metabolic gene sets also shifted downward with smaller magnitude (plasticity mean log2FC = −0.83; *p* = 8.1 × 10–9; trophic/metabolic mean log2FC = −0.58; *p* = 1.4 × 10–3; Supplementary Table S1). Because this dataset is whole-cochlea RNA, these signatures could reflect either within-neuron transcriptional regulation or cell-composition shifts; moreover, transcriptional suppression of excitability could be adaptive (dampening excitotoxic drive) or detrimental (reduced functional capacity). We therefore interpret directionality cautiously and frame these results as evidence that repair-oriented interventions can reshape programme-level signatures beyond simple survival metrics.

### 3.5 Construction of transparent CNFC modules is supported by functional enrichment

CNFC modules were constructed from literature-supported seed genes and then cross-checked/expanded using GO and KEGG enrichment of dataset-specific differentially expressed genes (Supplementary Table S1). This two-step process prioritizes interpretability while retaining an explicit link to unbiased functional annotation, and is intended to provide a hypothesis-generating scaffold rather than an exhaustive discovery of novel pathways.

### 3.6 A minimal 24-gene panel recapitulates full CNFC and generalises to independent SGNs

**Figure 9:**
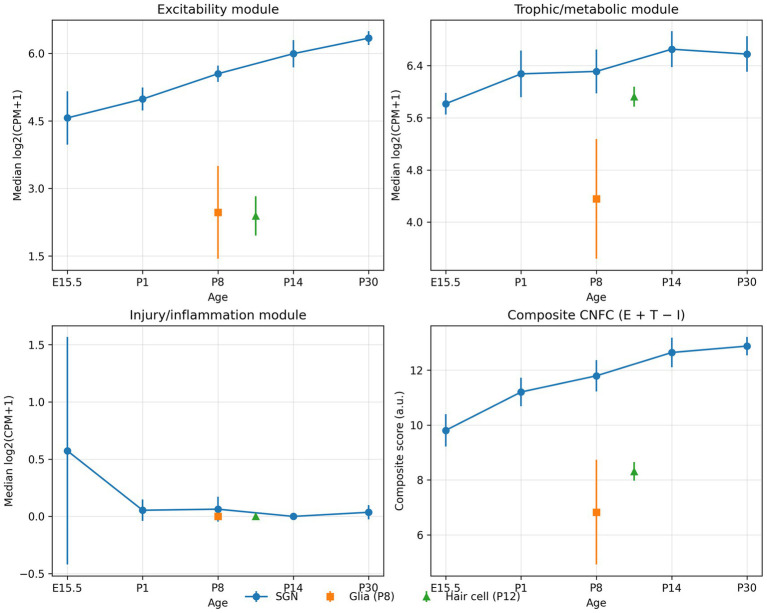
External validation of CNFC module trajectories in purified SGNs (GSE132925). Module scoring in an independent bulk RNA-seq dataset of purified spiral ganglion neurons (SGNs) across development (E15.5, P1, P8, P14, P30) with glial (P8) and hair-cell (P12) reference samples (GSE132925) (Li et al., 2020). For the minimal CNFC panel, module expression was summarised as the median log2(CPM + 1) across module genes, with points representing mean ± SD across biological replicates. Excitability increases with age in SGNs, trophic/metabolic support remains high, and injury/inflammation remains low, producing an increasing composite CNFC score (Excitability + Trophic/Metabolic − Injury/Inflammation).

Across the developmental FACS and adult microarray datasets, the minimal 24-gene panel closely tracked full-module CNFC scores (Pearson *r* = 0.83 within FACS; *r* = 0.86 within microarrays; overall *r* = 0.80; Figure 3B), supporting cross-platform portability. To assess generalisability beyond the primary datasets, we applied the minimal panel to an independent bulk RNA-seq dataset of purified mouse SGNs profiling five developmental ages (E15.5, P1, P8, P14 and P30) with glial (P8) and hair-cell (P12) reference samples (GSE132925) (Li et al., 2020). In purified SGNs, excitability increased with age, trophic/metabolic support remained high and injury/inflammation scores remained low, yielding an increasing CNFC trajectory from embryonic to mature stages (Figure 9). Glial and hair-cell reference samples showed substantially lower excitability and lower CNFC values than age-matched SGNs, supporting neuronal specificity of the competence trajectory.

### 3.7 CNFC aligns with an unbiased transcriptome-wide SGN maturity axis and is robust to alternative weighting

Principal-component analysis (PCA) of purified SGNs (GSE132925) on transcriptome-wide log2(CPM + 1) expression identified a dominant maturity axis (PC1 explained 45.3% variance). CNFC correlated strongly with PC1 across SGN samples (Pearson *r* = 0.94; Figure 4B), supporting that the curated CNFC modules capture a major unbiased transcriptional trajectory. To address potential non-equivalence of module weighting, we estimated data-driven coefficients by regressing PC1 on standardized module scores. The resulting weights emphasised excitability and trophic/metabolic support and down-weighted plasticity (Figure 4C). A weighted CNFC remained highly correlated with the unweighted composite (Pearson *r* = 0.97; Supplementary Table S1), supporting use of the simple interpretable formulation.

### 3.8 CNFC generalises to independent injury/degeneration transcriptomes beyond development

**Figure 10:**
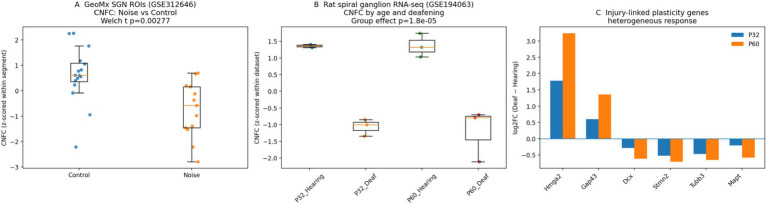
External injury/degeneration validation of CNFC. **(A)** GeoMx spatial transcriptomics of spiral ganglion regions after noise exposure (GSE312646): CNFC in neuronal ROIs shows a significant decrease in noise-exposed samples relative to controls (Welch *t*-test *p* shown). Module scores were computed using the intersection between the GeoMx panel and full CNFC gene sets and were *z*-scored within segment type. **(B)** Rat spiral ganglion RNA-seq after kanamycin-induced deafening (GSE194063): CNFC stratified by age (P32, P60) and hearing status. Group effect *p*-value is from a linear model including age as a covariate. **(C)** Selected plasticity-linked genes show a heterogeneous injury response in GSE194063: *Hmga2* and *Gap43* are induced in deafened spiral ganglia, whereas several neurite/cytoskeletal genes (*Dcx*, *Stmn2*, *Tubb3*) are reduced (log2FC = Deaf − Hearing). Boxplots show median and interquartile range; points represent biological replicates.

To test whether CNFC behaves meaningfully in independent injury/degeneration contexts (beyond the NIHL/MSC cohort and beyond developmental maturation), we analysed two additional public datasets not used for module construction: GeoMx spatial transcriptomics of spiral ganglion regions after noise exposure (GSE312646) and rat spiral ganglion RNA-seq after kanamycin-induced deafening (GSE194063) (Methods 2.4.1–2.4.2; Figure 10; Supplementary Table S1). In GeoMx neuronal ROIs, noise exposure significantly reduced excitability scores (*Δ* = −0.55 z units; *p* = 7.8 × 10–4) and lowered CNFC (*Δ* = −1.36; *p* = 2.8 × 10–3), with a trend toward higher injury/inflammation scores (*Δ* = +0.43; *p* = 0.051) despite limited gene-panel overlap. Plasticity scores also declined modestly (*Δ* = −0.32; *p* = 0.019). In the rat spiral ganglion dataset, hearing status (vs deafened) was associated with higher excitability (*β* = +0.79; *p* = 0.005) and trophic/metabolic support (*β* = +0.62; *p* = 6.2 × 10–4), lower injury/inflammation (*β* = −1.09; *p* = 0.0015), and a robust increase in CNFC (*β* = +2.51; *p* = 1.8 × 10–5) after controlling for age. Gene-level inspection revealed a heterogeneous plasticity response: growth-associated regulators (Hmga2, Gap43) were induced in deafened spiral ganglia, whereas several neurite/cytoskeletal genes (Dcx, Stmn2, Tubb3) were reduced (Figure 10C), supporting partial and non-uniform re-engagement of plasticity programmes after injury.

## 4 Discussion

### 4.1 From structural survival to functional competence

A long-standing clinical intuition is that cochlear implant performance depends on how many auditory neurons survive after deafness. However, the relationship between spiral ganglion survival and speech recognition is variable and often modest (Cheng and Svirsky, 2021), and prediction of speech outcomes remains difficult even when multiple clinical factors are considered (Bartholomew et al., 2024). Our integrative analysis supports the concept that a functional competence state—encompassing excitability, synaptic signalling and plasticity programmes—may be an important latent variable. Under this view, neurons (and the surrounding cochlear microenvironment) can remain structurally present while functionally compromised in their ability to encode temporally precise information and to adapt to chronic electrical stimulation.

### 4.2 Excitability programmes and temporal coding

Auditory perception requires rapid, phase-locked firing and high-rate spiking. Developmental acquisition of these properties is known to involve fast potassium channels, voltage-gated sodium channels, and coordinated synaptic maturation (Boulet et al., 2016; Wang et al., 1998; Adamson et al., 2002; Hossain et al., 2005; Browne et al., 2017). Within the EGFP- fraction, we observed developmental increases in glutamatergic receptor genes such as Gria1 and in a subset of fast-spiking channel genes such as Kcnc1 (Figure 7). Although the EGFP- fraction is heterogeneous and cannot be equated with purified SGNs, these signatures are consistent with maturation of a neural transcriptome component within the developing cochlea. In the NIHL dataset, MSC therapy suppressed multiple excitability-related genes (including Grin2a/Grin2c, Kcnc1 and Scn8a) (Figure 2), suggesting that post-injury interventions may act, at least in part, by rebalancing excitability and excitotoxicity pathways implicated in synaptopathy (Kujawa and Liberman, 2009; Lin et al., 2011; Liberman and Kujawa, 2017).

### 4.3 Plasticity programmes and electrode-neuron coupling

A major barrier to improving CI outcomes is the physical distance between electrode contacts and target neural processes (Finley et al., 2008; Holden et al., 2016). In principle, neurite regeneration toward the electrode array could tighten electrode–neuron coupling and reduce stimulation thresholds. Our developmental analysis highlights a robust decline of actin-remodelling and growth-associated programmes (e.g., Cfl1/Cfl2 and Hmga2), consistent with a postnatal consolidation of structural plasticity. Importantly, developmental down-regulation does not imply that all plasticity-linked genes are uniformly silent after injury: in the rat kanamycin-deafening dataset (GSE194063) we observed selective induction of growth-associated regulators (Hmga2, Gap43) alongside suppression of several neurite/cytoskeletal genes (Dcx, Stmn2, Tubb3) (Figure 10C; Supplementary Table S1). This mixed pattern is consistent with an attempted but constrained regenerative response, and reinforces the rationale for interventions that both re-activate growth programmes and preserve excitability/synaptic competence—such as neurotrophin delivery or modulation of growth-cone signalling (Ramekers et al., 2012; Wan et al., 2014; Suzuki et al., 2016; Wang and Green, 2011).

### 4.4 Sensory-epithelial trophic support as a modifiable determinant of neural competence

Neurons do not exist in isolation: the sensory epithelium and adjacent supporting cells provide neurotrophic ligands, extracellular matrix scaffolds, and metabolic buffering that shape neuronal resilience (Ramekers et al., 2012; Nayagam et al., 2011; Reijntjes and Pyott, 2016). The adult IHC/OHC microarray analysis emphasised IHC-biased expression of Ntf3 and Gdnf (Figure 8; Liu et al., 2014), aligning with the critical role of hair-cell-derived neurotrophin signalling in maintaining type I SGN innervation and in promoting synaptic repair after injury (Ramekers et al., 2012; Wan et al., 2014; Suzuki et al., 2016; Wang and Green, 2011). These observations motivate a microenvironment-centric view of cochlear neural health, where preserving (or replacing) sensory-epithelial trophic support may protect functional competence even when hair-cell transduction is lost.

### 4.5 Limitations and future directions

This study has several important limitations. First, the developmental dataset is based on FACS-separated hair cells and a heterogeneous EGFP− fraction rather than purified SGNs; consequently, EGFP− trajectories likely reflect both transcriptional maturation and changing cellular composition. We partially addressed this with cell-type signature scoring (Figure 6), but single-cell or nucleus-resolved approaches will be needed for a more direct SGN subtype-specific framework.

Second, CNFC modules are intentionally curated and mechanistically interpretable rather than derived solely from unsupervised clustering. We adopted a curated approach because our primary goal is a portable scoring vocabulary that can be applied across heterogeneous cochlear studies and platforms; in small or mixed-cell datasets, unsupervised modules (e.g., WGCNA) can be sensitive to sample size, cell-type composition and technical covariates and may not map one-to-one across studies or species. Accordingly, CNFC should be viewed as a hypothesis-generating scaffold. To reduce subjectivity, we provide transparent gene lists (Supplementary Table S1), enrichment support, an unbiased transcriptome-wide benchmark (PCA in purified SGNs; Figure 4), and sensitivity analyses for weighting and scoring.

Third, because datasets were generated on different platforms and in different contexts, we avoid direct pooling or aggressive batch correction and rely on within-dataset standardized scoring; absolute CNFC values should therefore not be compared across studies without harmonised processing. Finally, while we demonstrate CNFC behaviour across multiple injury/degeneration contexts (NIHL ± MSC, GeoMx NIHL, and kanamycin deafening), the present work remains based on animal transcriptomes and does not include patient-level CI outcomes. Future studies should integrate larger SGN-resolved injury/regeneration datasets and test whether CNFC-like scores derived from clinically accessible biospecimens (e.g., perilymph) prospectively associate with CI performance.

### 4.6 Testable predictions and translational roadmap

Our analysis is intended to generate testable, mechanistically anchored hypotheses rather than to over-interpret public datasets. Within that spirit, the CNFC framework suggests several concrete predictions that can be evaluated experimentally or in clinical cohorts:

(1) Patients (or animal models) with higher pre-intervention excitability/synaptic module scores—measured directly in SGN tissue where available, or inferred from proximal fluids such as perilymph—will show stronger electrically evoked responses and better speech recognition after CI.
(2) Elevated injury/inflammation module activity at the time of implantation (or after acoustic trauma) will correlate with poorer neural coding and reduced benefit, motivating anti-inflammatory or glia-targeted adjunct therapies to preserve competence.
(3) Sensory-epithelial trophic/metabolic support signatures (e.g., neurotrophins and oxidative-stress buffering genes) will associate with resilience of the auditory nerve to chronic stimulation and with long-term outcome stability.
(4) A minimal CNFC panel (Table 3; Supplementary Table S1) can be implemented as a targeted assay (e.g., qPCR/ddPCR or targeted RNA-seq) in future studies to enable rapid competence profiling and cross-cohort comparability.

Recent perilymph biomarker studies and systematic reviews support the feasibility of sampling cochlear fluids and linking molecular states to CI outcome variability (Wichova et al., 2021; Wanniarachchi et al., 2025). By providing an explicit module-based vocabulary and an initial minimal gene panel, our framework aims to accelerate the design of such validation studies and to connect cellular neuroscience mechanisms to clinically actionable neuroprosthetic hearing phenotypes.

## 5 Conclusion

By integrating developmental, adult and injury-related cochlear transcriptomes, we propose a molecular framework for cochlear neural functional competence built around four programme classes: excitability/synaptic transmission, cytoskeletal plasticity, sensory-epithelial trophic-metabolic support and injury-response modulation. This framework may help explain why anatomical survival metrics are often insufficient to predict cochlear implant outcomes and generates testable hypotheses for biomarker discovery and therapeutic strategies aimed at preserving or restoring neural function.

## Data Availability

The original contributions presented in the study are included in the article/Supplementary material, further inquiries can be directed to the corresponding authors.
